# Development of gemcitabine-resistant patient-derived xenograft models of pancreatic ductal adenocarcinoma

**DOI:** 10.20517/cdr.2020.35

**Published:** 2020-08-07

**Authors:** Aubrey L. Miller, Patrick L. Garcia, Tracy L. Gamblin, Rebecca B. Vance, Karina J. Yoon

**Affiliations:** Department of Pharmacology and Toxicology, University of Alabama at Birmingham, Birmingham, AL 35294 USA.

**Keywords:** Gemcitabine resistance, patient-derived xenograft, ribonucleotide reductase subunit M1, ribonucleotide reductase subunit M2, human concentrative nucleoside transporter 1, human equilibrative nucleoside transporter 1, cytidine deaminase, deoxycytidine kinase

## Abstract

**Aim**: Gemcitabine is a frontline agent for locally-advanced and metastatic pancreatic ductal adenocarcinoma (PDAC), but neither gemcitabine alone nor in combination produces durable remissions of this tumor type. We developed three PDAC patient-derived xenograft (PDX) models with gemcitabine resistance (gemR) acquired *in vivo*, with which to identify mechanisms of resistance relevant to drug exposure *in vivo* and to evaluate novel therapies.

**Methods**: Mice bearing independently-derived PDXs received 100 mg/kg gemcitabine once or twice weekly. Tumors initially responded, but regrew on treatment and were designated gemR. We used immunohistochemistry to compare expression of proteins previously associated with gemcitabine resistance [ribonucleotide reductase subunit M1 (RRM1), RRM2, human concentrative nucleoside transporter 1 (hCNT1), human equilibrative nucleoside transporter 1 (hENT1), cytidine deaminase (CDA), and deoxycytidine kinase (dCK)] in gemR and respective gemcitabine-naïve parental tumors.

**Results**: Parental and gemR tumors did not differ in tumor cell morphology, amount of tumor-associated stroma, or expression of stem cell markers. No consistent pattern of expression of the six gemR marker proteins was observed among the models. Increases in RRM1 and CDA were consistent with *in vitro*-derived gemR models. However, rather than the expected decreases of hCNT1, hENT1, and dCK, gemR tumors expressed no change in or higher levels of these gemR marker proteins than parental tumors.

**Conclusion**: These models are the first PDAC PDX models with gemcitabine resistance acquired *in vivo*. The data indicate that mechanisms identified in models with resistance acquired *in vitro* are unlikely to be the predominant mechanisms when resistance is acquired *in vivo*. Ongoing work focuses on characterizing unidentified mechanisms of gemR and on identifying agents with anti-tumor efficacy in these gemR models.

## Introduction

Pancreatic cancer remains one of the few solid tumors with a five-year survival less than 10%^[1]^. Pancreatic ductal adenocarcinoma (PDAC) accounts for > 90% of pancreatic cancers^[2]^. Most PDAC patients present with locally advanced or metastatic disease, prognostic indicators for unfavorable outcome. Surgical resection is potentially curative, but few patients present with resectable disease. For most patients with nonresectable disease, gemcitabine-based therapies have been standard of care agents for more than two decades^[3-5]^.

Gemcitabine [difluorodeoxycytidine (dFdC)] is a pyrimidine nucleoside analog and prodrug with multiple mechanisms of action. Phosphorylation by deoxycytidine kinase (dCK), a rate-limiting enzyme of gemcitabine metabolism, activates dFdC intracellularly by converting the parent molecule to its mono- [dFdC monophosphate (dFdCMP)], di- [dFdC diphosphate (dFdCDP)] and tri- [dFdC triphosphate (dFdCTP)] phosphate forms^[6]^. The principal mechanism of action of gemcitabine is thought to involve competition with natural nucleotides by dFdCTP, incorporation of dFdCTP into DNA, inhibition of DNA polymerase, and production of dysfunctional DNA resulting in apoptosis^[7]^. Despite clinical utility, resistance frequently develops within weeks or months after initiation of treatment^[8,9]^. Numerous studies focused on identifying molecular bases for gemcitabine resistance have been equivocally successful in designing effective therapies. One of the latest reports by Cascioferro *et al.*^[10]^ documented an interesting note that some of imidazo [2,1-b][1.3.4] thiadiazole derivatives showed low micromolar antiproliferative activity against gemcitabine resistant Panc-1 pancreatic cancer cells (Panc-1R) *in vitro*. The authors also showed that a potential mechanism of these derivatives could be inhibition of phosphorylation of PTK2/FAK.

Reported mechanisms of gemcitabine-resistance (gemR) include limited gemcitabine uptake due to decreased expression of nucleoside transporters such as human concentrative nucleoside transporter (hCNT) or human equilibrative nucleoside transporter (hENT)^[11-13]^; limited activation due to decreased expression of the activating enzyme dCK and increased expression of the deactivating enzyme cytidine deaminase (CDA)^[14-16]^; increased stroma: tumor cell ratio^[6,8,17]^; increased expression of proteins that support survival or proliferation such as proteins of the PI3K/AKT pathway^[18,19]^ or stem cell marker proteins such as CD133 and ALDH1^[20-22]^; and increased activity of anti-apoptotic proteins such as Bcl-xL and Bax^[23,24]^. Work characterizing gemcitabine resistance mechanisms has been done primarily with *in vitro* cell lines and cell line-based xenograft models. We developed patient-derived xenograft (PDX) models derived from primary human tumors with resistance acquired *in vivo*, as tools to identify which resistance mechanisms in cell line-based models also occur in models designed to reflect *in vivo* responses to gemcitabine. These models comprise tools with which to identify additional mechanisms of resistance and to evaluate novel treatment regimens. We and others have shown that PDX models retain specific genotypic and phenotypic characteristics of the primary tumors from which they were derived^[25,26]^. Notably, this type of model has also predicted clinical response to several therapeutic agents including the combination of gemcitabine + nab-paclitaxel^[27-29]^. We suggest that PDX models in which resistance to gemcitabine is acquired *in vivo* may more closely reflect clinical responses to this agent than cell line-based models.

The goal of this study was to establish, for the first time, acquired gemR PDX models of PDAC, and to compare the morphology and histology of gemR and gemcitabine-sensitive parent models, and to assess immunohistochemistry (IHC) expression of nucleoside transport proteins (hCNT1, hENT1), nucleoside metabolizing enzymes (RRM1, RRM2, CDA, dCK) and stem cell markers (ALDH1, CD133, CXCR4).

## Methods

### Generation of gemcitabine-resistant PDX models

Previously established UAB-PA4, UAB-PA10 and UAB-PA16 PDX tumors^[25]^ were implanted subcutaneously into four- to six-week old female SCID CB 17^-/-^ mice that were purchased from Taconic farms (Newton, MA, USA) or Charles River (Wilmington, MA, USA). All animals were housed in the AAALAC accredited vivarium at UAB Research Support Building under barrier conditions with a 12-h light/dark cycle and access to food and water *ad libitum*. When tumors reached 200-800 mm^3^ in size mice were randomized into two treatment cohorts: Control (*n* = 4-12) or gemcitabine (*n* = 3-10). Mice bearing bilateral tumors received i.p. injections of gemcitabine (100 mg/kg) weekly (UAB-PA10 and -PA16) or biweekly (UAB-PA4) until the tumors no longer responded to treatment, determined by increases in tumor volume. Control groups of mice bearing UAB-PA10 or -PA16 tumors received i.p. saline injections weekly. Control mice bearing UAB-PA4 tumors were untreated. Tumors were measured three times a week using digital calipers, and tumor volume was calculated using the formula v= (π/6)*d^3^. Tumor volumes were compared weekly with tumor volumes at day one of the treatment period, and an increase of > 20% was interpreted as tumor growth. During the gemR development period, all mice maintained their body weights and no visible toxicity was observed. When tumor regrowth was confirmed, tumor tissue was harvested and transplanted for propagation *in vivo*, frozen as viable tissue, snap frozen, or formalin-fixed and embedded in paraffin.

### Gemcitabine efficacy study in PDX models

Four- to six-week old CB 17^-/-^ female SCID mice were purchased from Taconic Farms (Newton, MA, USA) and housed as described above. Mice bearing UAB-PA4, -PA10 or -PA16 tumors were randomized into two groups of 4-5 mice/group when tumors reached ~200 mm^3^. The control group of mice received saline twice weekly, and the gemcitabine group received 100 mg/kg gemcitabine twice weekly for 3 weeks. Gemcitabine was purchased from LC Laboratories (G-4177, Woburn, MA, USA), and gemcitabine solutions were prepared immediately prior to injection. Tumor size was measured twice weekly using digital calipers as described above. Tumor volumes are presented as mean ± S.E.M.

### Hematoxylin and Eosin staining

Hematoxylin and eosin (H&E) staining was performed as previously described^[25]^. Photomicrographs were taken with an Olympus BH-2 microscope with DP71 camera and DPS-BSW v3.1 software (Center Valley, PA, USA).

### Immunohistochemistry

Immunohistochemical staining and analysis was performed as previously published^[30-32]^. Briefly, Ki67 proliferation indices were determined by counting the number of Ki67 positive tumor cells in 10 individual 40x fields and dividing that number by the total number of tumor cells. The data are expressed as the mean ± S.E.M. of two independent experiments. Expression indices of RRM1, RRM2, hENT1, hCNT1, CDA, dCK, ALDH1, CD133, and CXCR4 were calculated by assigning a staining intensity of 0, 1, 2, or 3 to each specimen, and multiplying this intensity by the percent of tumor cells expressing the protein of interest^[33]^. Overall scores ranged from 0 to 300. Data were derived from photomicrographs taken with Olympus BH-2 microscope with DP71 camera and DPS-BSW v3.1 software. Primary antibodies were obtained from multiple sources: Ki67 (ab92742, abcam, Cambridge, MA, USA), RRM1 (NBP2-49415, Novus Biologicals, Littleton, CO, USA), RRM2 (ab57653, abcam), hENT1 (SAB5500117, Sigma-Aldrich, St. Louis, MO, USA), hCNT1 (NBP2-30857, Novus Biologicals), CDA (ab82346, abcam), dCK (sc393099, Santa Cruz, Dallas, TX, USA), ALDH1 (sc166362, Santa Cruz), CD133 (CS#86781T, Cell Signaling, Danvers, MA, USA), CXCR4 (TA305935, Origene, Rockville, MD, USA).

### *KRAS* mutational analysis

Detailed methods were published previously^[25]^. Briefly, genomic DNA was isolated from UAB-PA4, -PA10, and -PA16 parent tumors and gemcitabine resistant counterparts using a DNA/RNA extraction kit (EpiCentre, Madison, WI, USA). DNA extracted from human Panc1 pancreatic cancer cells was the positive control and DNA from mouse liver was a negative control. Panc1 cells were purchased from the American Type Culture Collection (Manassas, VA, USA). Concentration and quality of DNA was determined using a ND-1000 spectrophotometer and Nanodrop 3.0.1 software (Nanodrop, Coleman Technologies, Inc., Wilmington, DE, USA). PCR reactions were performed with 400 ng of DNA and the previously published human-specific *KRAS* primers (F: 5’gtgtgacatgttctaatatagtca3’ and R:5’gaatggtcctgcaccagtaa3’)^[25]^. These primers generated a 214bp PCR product that included codons 12 and 13 in exon 2, each of which is commonly mutated in PDAC tumors. Gel electrophoresis was performed with 2% agarose gel, the 214bp product extracted using a gel purification kit (Fisher Scientific, Waltham, MA, USA), and sequencing performed by the Heflin Center for Genomic Sciences (University of Alabama at Birmingham, Birmingham, AL, USA). All PCR products were sequenced twice in the forward direction. Electropherograms were analyzed using FinchTV (version1.4.0; www.geospiza.com).

### Statistical analysis

All statistics were performed using GraphPad Prism v7.0 or v8.0. Tumor volumes were compared using two-way analysis of variance (ANOVA), and Ki67 proliferation indices were compared by Student’s *t*-test. *P* < 0.05 was considered significant.

## Results

### Generation of gemR PDX models

**Figure 1:**
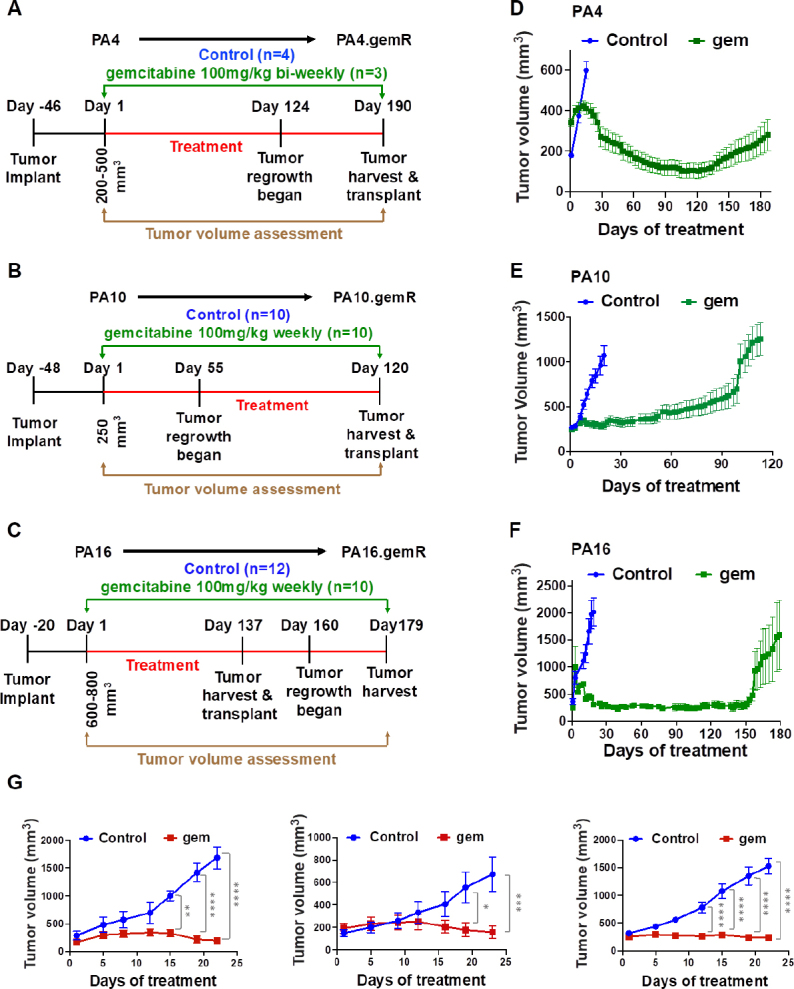
Development of acquired gemcitabine resistance in three PDX models of PDAC. Schematic representation of *in vivo* gemR model development. The schematic shows drug regimen for gemcitabine and times to development of acquired gemcitabine resistance for PA4 (A), PA10 (B), and PA16 (C) PDX models of PDAC. The number (*n*) of tumors in each cohort is shown in parentheses (A-C); tumor growth curves illustrating changes in sensitivity to gemcitabine in 3 PDX models. Gemcitabine was given once or twice weekly from days 1 through 120-190, as indicated by day of tumor harvest (D-F); tumor growth curves for parental PA4, PA10 and PA16 models (*n* = 4-5 tumors/cohort) (G). Mice were treated twice weekly with 100 mg/kg gemcitabine or with saline (control) for three weeks. All tumor volumes are reported as mean ± S.E.M. Tumor volumes were compared using two-way analysis of variance (ANOVA) (*P < 0.05, **P < 0.01, ***P < 0.001, ****P < 0.0001). PDAC: pancreatic ductal adenocarcinoma; PDX: patient-derived xenograft

Our lab previously published development and characterization of parental UAB-PA4, UAB-PA10 and UAB-PA16 PDAC PDX models^[25]^, hereafter referred to as PA4, PA10 and PA16. As previously reported, all tumors were stage II and were moderately to poorly differentiated^[30]^. The patients from whom tumor tissue was obtained had evidence of lymph node involvement at time of resection. All three primary tumors harbored a mutation in codon 12 of the *KRAS* gene, a mutation common in PDAC tumors. This was conserved in the PDX models^[25]^. A schematic showing treatment and dosing schedules used to develop each resistant model is shown in Figure 1A-C.

All three parent models were initially sensitive to gemcitabine, however, within 7-19 weeks, tumor progression became evident on therapy [Figure 1A-F]. Mice bearing PA4 tumors were treated with gemcitabine 100 mg/kg biweekly [Figure 1A]. When tumors reached ~300 mm^3^, treatment with gemcitabine started. The control group of PA4 was untreated. Mice remained on gemcitabine treatment until the tumors no longer responded to treatment and were considered gemcitabine resistant when the tumor volume increased while on treatment. Following 120 days of gemcitabine treatment PA4 tumor volume had reduced 80% the maximum tumor volume, indicating a partial response as defined by ≥ 50% reduction in tumor volume for at least one time point ^[34]^, and regrowth of the tumor began shortly thereafter at 124 days of treatment [Figure 1A and D]. When PA10 tumors reached ~250 mm^3^, cohorts of mice were treated with vehicle control (saline) or gemcitabine 100 mg/kg weekly [Figure 1B]. While PA10 tumors did not regress on gemcitabine treatment, stable disease was maintained in the mice for > 50 days. Regrowth began around day ~55 of treatment, but it was a slow growth phenotype until day 100 when the tumor growth rate appeared to increase [Figure 1B and E]. Mice bearing PA16 began gemcitabine 100 mg/kg weekly when tumor volume reached ~600 mm^3^. Similar to PA4, PA16 tumor volume was also reduced by 80% following 150 days of gemcitabine treatment and tumor regrowth was evident at ~160 days of treatment [Figure 1C and F]. Although the majority of PA16.gemR tumors were developed after ~160 days of treatment, we observed that some tumors regrew faster than others (day ~137) [Figure 1C]. Further, we assessed gemcitabine sensitivity using PA4, PA10 and PA16 parent PDX models. As shown in Figure 1G, all three parent models were sensitive to gemcitabine (100 mg/kg twice weekly), as quantitated by differences in tumor volumes of treated animals compared to controls (*P* < 0.05).

### All three gemR models showed increased levels of proliferation marker Ki67

**Figure 2:**
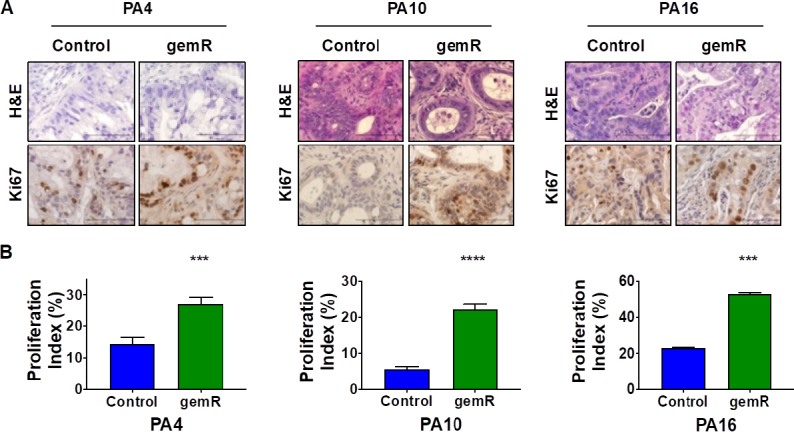
All gemR models showed increased levels of proliferation marker Ki67. Tumor tissue harvested on days 190, 120, and 179 for PA4.gemR, PA10.gemR and PA16.gemR, respectively, was stained with H&E to visualize tumor and stroma morphology, and was immunostained for the proliferation marker Ki67. Tumor tissue harvested from control mice bearing PA4, PA10 or PA16 tumors (gemcitabine-naïve) served as controls for IHC staining experiments. Scale bar = 10 μm (A); data in ‘A’ were quantitated to determine Ki67 proliferation indices (%). Bar graphs show mean ± S.E.M. Ki67 expression. Data were analyzed using Student’s *t*-test (***P < 0.001, ****P < 0.0001) (B). gemR: gemcitabine resistance; IHC: immunohistochemistry

We next assessed whether long-term gemcitabine treatment affected tumor cell morphology or histology. In *in vitro* models of acquired gemcitabine resistance, cancer cells have shown an invasive fibroblast-like phenotype^[35]^. Further, expression array analysis showed an increase in stroma-related pathways in gemcitabine resistant tumors as well as an increase in stroma:tumor cell ratio *in vivo*^[17]^. H&E stained formalin-fixed paraffin-embedded (FFPE) sections from tumors harvested at days 190, 120, and 179 for PA4.gemR, PA10.gemR and PA16.gemR, respectively, showed no change in morphology, degree of differentiation, or tumor:stroma ratio compared to their drug sensitive counterparts (control) [Figure 2A].

We next examined whether increases in tumor volume were concomitant with increases in proliferation indices. Immunohistochemistry (IHC) staining for the proliferation marker Ki67 demonstrated that Ki67 levels of PA4.gemR, PA10.gemR, and PA16.gemR tumors were higher than their control counterparts (*P* < 0.001) [Figure 2A and B].

### Characterization of models selected for gemcitabine resistance *in vivo* for expression of six proteins demonstrated to be altered in models developed *in vitro*

**Figure 3:**
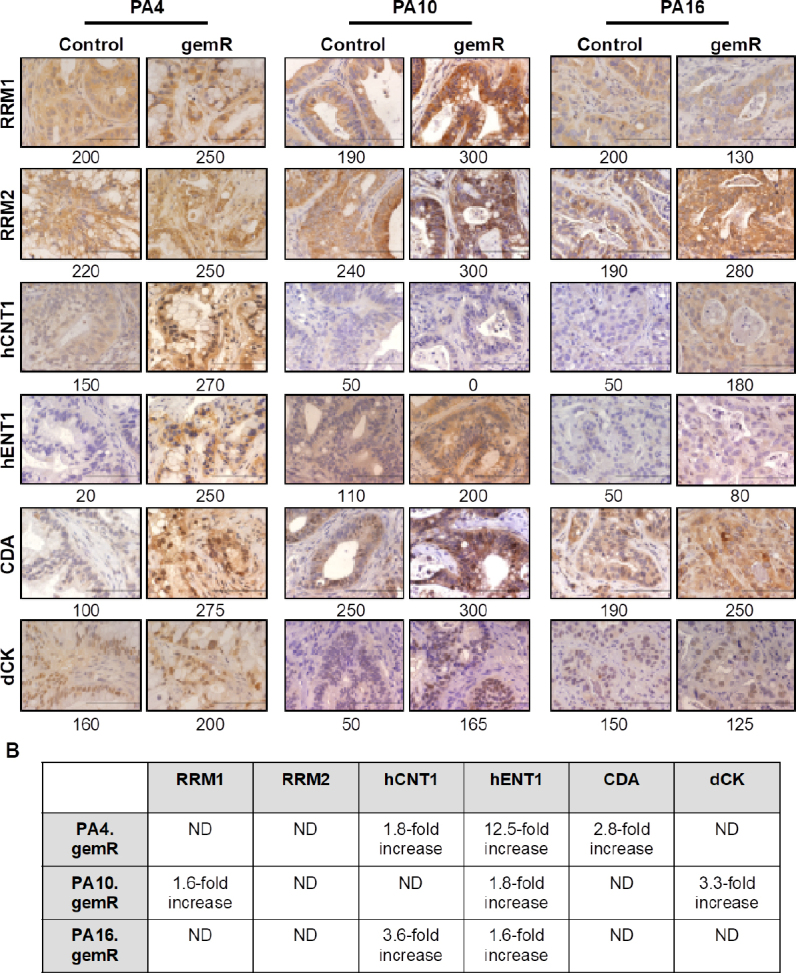
PDAC PDX models of acquired gemcitabine resistance harbored changes in expression of some gemcitabine transporters and metabolizing enzymes. Tumor tissue harvested on days 190, 120, and 179 for PA4.gemR, PA10.gemR and PA16.gemR, respectively, was immunostained for RRM1, RRM2, hCNT1, hENT1, CDA, and dCK. Tumor tissue harvested from control mice bearing gemcitabine-naïve PA4, PA10 or PA16 tumors served as controls for IHC staining experiments. Scale bar = 10 μm. Quantitation of IHC data in A is shown as expression indices below each photomicrograph (A); protein expression in gemR models that were developed by exposure to drug *in vivo*, compared to their respective parent tumor models. Each protein has been reported to be associated with gemcitabine resistance in models developed *in vitro*. ND: no difference between gemcitabine-naïve control and gemR tumors. Values in the Table were calculated by dividing the EI of each protein determined by IHC expressed by gemR/parent (control) tumors. A change of > 1.5-fold was interpreted as a real increase or decrease (B). PDAC: pancreatic ductal adenocarcinoma; PDX: patient-derived xenograft; gemR: gemcitabine resistance; IHC: immunohistochemistry

We next assessed whether the phenotype of the gemR models acquired *in vivo* differed from drug-sensitive counterparts with respect to expression of proteins involved in gemcitabine transport into tumor cells (hCNT1 and hENT1) or in gemcitabine metabolism (RRM1, RRM2, CDA, dCK) [Figure 3].

We first assessed levels of expression of ribonucleotide reductase subunits M1 and M2 (RRM1 and RRM2), which contribute to regulating levels of intracellular nucleotides. The diphosphate form of gemcitabine inhibits these enzymes and subsequent incorporation of dCTP into DNA^[36]^. Published data demonstrate that increases in RRM1 or RRM2 are associated with gemcitabine resistance *in vitro*^[8,37-39]^. However, of the three gemR PDX models, only PA10.gemR showed a 1.6-fold increase in RRM1 expression [Figure 3A and B]. We next assessed levels of expression of two transporter proteins that mediate cellular uptake of gemcitabine, hCNT1 and hENT1. In *in vitro*-based models, decreased expression of these transporters and therefore decreased concentrations of intracellular gemcitabine is associated with gemcitabine resistance^[11,40-42]^. Inconsistent with *in vitro* data, all three models expressed increased, rather than decreased, levels of hENT1 (1.6- to 12.5-fold) and two models expressed increased levels of hCNT1 (1.8- to 3.6-fold) [Figure 3A and B]. Thirdly, we assessed expression of the gemcitabine metabolizing enzymes CDA and dCK, which regulate levels of the active forms of gemcitabine. CDA deaminates parent gemcitabine (dFdC) to produce inactive difluorodeoxyuridine (dFdU)^[36-38]^. dCK phosphorylates gemcitabine, a rate-limiting step in converting gemcitabine to its active form. Consistent with these metabolic functions, an increase in CDA and/or a decrease in dCK is associated with gemcitabine resistance *in vitro*^[43-46]^. Consistent with *in vitro* data, IHC staining showed an increase in CDA expression in a single model, PA4.gemR. In contrast, and again inconsistent with *in vitro* data, PA10.gemR showed a 3.3-fold increase rather than a decrease in dCK [Figure 3A and B]. We also noted that no pattern in changes of expression of the six marker proteins was common to all three models. The data suggest that PDAC PDX models with gemcitabine resistance acquired *in vivo* harbor resistance mechanisms not previously identified in models with resistance developed *in vitro*. The data suggest further that because changes in expression of a given protein did not occur concomitantly with any other marker protein, it is likely that each change developed independently.

### No consistent changes were observed in expression of stem cell markers in gemR models

**Figure 4:**
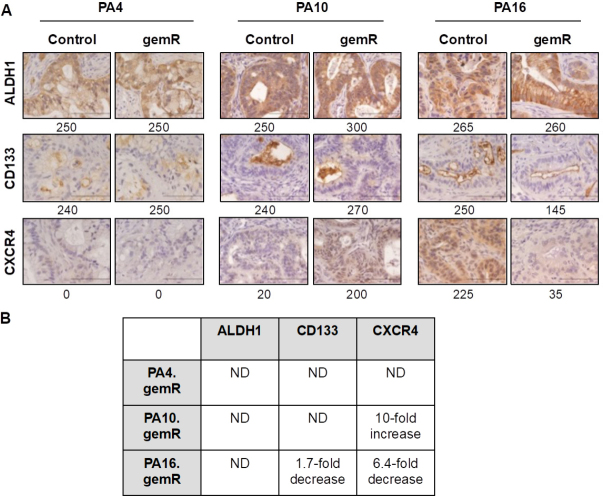
No consistent changes were observed in expression of stem cell markers in gemR models compared to drug-sensitive controls. IHC analysis of ALDH1, CD133, and CXCR4 in PA4, PA10, and PA16 control and gemR tumors. Tumor tissue harvested on days 190, 120, and 179 for PA4.gemR, PA10.gemR and PA16.gemR, respectively, was used for IHC analyses. Tumor tissue harvested from gemcitabine-naïve control mice bearing PA4, PA10 or PA16 tumors served as controls. Scale bar = 10 μm. Expression indices of IHC data are shown below each photomicrograph (A); fold changes in protein expression in gemR models compared to their respective parent models. ND = no difference between gemcitabine-naïve control and gemR tumors. Values in the Table were calculated by dividing the EI of each protein determined by IHC expressed by gemR/parent tumor. A change of > 1.5-fold was interpreted as a real increase or decrease (B). gemR: gemcitabine resistance; IHC: immunohistochemistry

Pancreatic cancer stem cells are defined as the subset of cells in a tumor that express specific marker proteins. Tumor cells that express these markers have been characterized in several studies as chemoresistant to agents that include gemcitabine, 5-fluorouracil, and nab-paclitaxel^[47,48]^. Unlike the involvement of specific gemcitabine transporters and metabolizing enzymes, stem cell markers are thought to be involved in resistance to multiple classes of agents in addition to antimetabolites such as gemcitabine. To address the hypothesis that the gemR phenotype was associated with tumor cell stemness, we characterized the expression of stem cell markers ALDH1, CD133, and CXCR4 in gemR tumors [Figure 4]^[20,22,49]^. *In vitro* studies using an siRNA approach have shown ALDH1 confers up to ~4.5-fold resistance to gemcitabine in PDAC cells^[49]^. However, IHC staining showed no difference in levels of ALDH1 between gemR tumors and gemcitabine-naïve controls [Figure 4A and B]. These data suggest that stemness, as assessed by expression of ALDH1, was not associated with the *in vivo* gemR phenotype. *In vitro* studies have also demonstrated that PDAC cell lines expressing CD133 were resistant to gemcitabine^[20]^, but our PA16.gemR model showed a decrease rather than an increase in CD133 positive cells [Figure 4A and B]. A third reported PDAC cancer stem cell marker, CXCR4, has also been associated with drug resistance^[22]^, but again there was no consistent pattern of increased or decreased expression of this marker protein in gemR tumor models. IHC data did show a 10-fold increase in CXCR4 in PA10.gemR tumors and a 6.4-fold decrease in this marker in PA16.gemR tumors, compared to controls [Figure 4A and B].

Overall, the data demonstrated no consistent alteration of expression of the cancer stem cell markers evaluated and no association of expression of these markers with the gemR phenotype. In contrast to the reported association of tumor cell stemness with a gemcitabine resistant phenotype *in vitro*, the data did not support the hypothesis that the *in vivo* gemR phenotype is linked to tumor cell stem-like properties.

### Gemcitabine resistance was maintained in the PA16.gemR tumors in the absence of gemcitabine treatment for greater than 5 weeks

**Figure 5:**
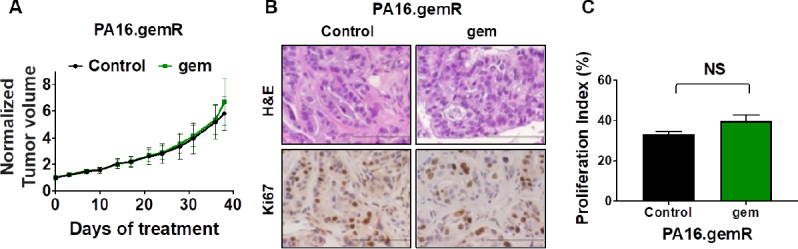
PA16.gemR tumors maintained gemcitabine resistant phenotype in the absence of exposure to gemcitabine. Average tumor volumes ± S.E.M. of PA16.gemR tumors in mice treated with gemcitabine 100 mg/kg weekly or with saline control for 5 weeks. Tumor volumes are normalized to day 0 of treatment (A); photomicrographs of PA16.gemR tumors harvested three days after the completion of therapy were stained for H&E and immunostained for the proliferation marker Ki67. Scale bar = 10 μm (B); Ki67 IHC data in B were quantitated as described in Methods to determine proliferation indices. Ki67 proliferation indices were compared by Student’s *t*-test. NS: not significant (C). gemR: gemcitabine resistance; IHC: immunohistochemistry

To address whether the gemR phenotype was maintained *in vivo* in the absence of gemcitabine, PA16.gemR tumor specimens were implanted bilaterally and propagated *in vivo* in the absence of gemcitabine treatment for ~5 weeks, at which time tumor volumes reached ~200 mm^3^. One cohort of mice was then treated with gemcitabine 100 mg/kg weekly for 5 weeks and one cohort treated with saline (control). Tumor response was the same in mice treated with gemcitabine compared to saline [Figure 5A]. The data indicate that tumors retained resistance to gemcitabine in the absence of exposure to gemcitabine for greater than the 5-week period during which they were not exposed to gemcitabine. Three days after the final weekly drug treatment, tumors were harvested, and FFPE sections made. H&E stained sections of saline-treated PA16.gemR tumors showed similar tumor morphology and degree of differentiation as gemcitabine-treated PA16.gemR tumors [Figure 5B, H and E]. Immunohistochemistry for the proliferation marker Ki67 also showed similar proliferation indices in tumors from gemcitabine- and saline-treated mice [Figure 5B and C]. The data demonstrate that gemR tumors maintain their resistant phenotype *in vivo* for > 5 weeks. Of note, when gemR models are used in efficacy testing, we will use these models in the presence of gemcitabine.

### Each gemR PDX model retained the KRAS mutation status of the drug-sensitive parent model from which it was derived

**Figure 6:**
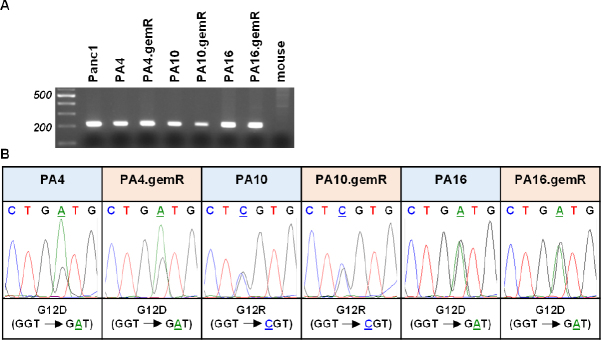
gemR PDX models retained mutations in codon 12 of the *KRAS* gene that were present in parent PDX models. Human-specific primers amplified the expected 214 base pair PCR product that included codons 12 and 13 of exon 2 of the *KRAS* gene. DNA from mouse tissue was used as a negative control (A); electropherograms document retention by gemR PDX models of mutations in codon 12 of the *KRAS* gene that are present in respective parent models (B). gemR: gemcitabine resistance; PDX: patient-derived xenograft

PDX tumor models have been documented to retain specific genetic and molecular characteristics of their primary tumors of origin. In PDAC tumors one of these characteristics is a mutation in *KRAS* of codon 12 or 13, both in exon 2. We previously reported using human-specific KRAS primers to confirm that PA4, PA10, and PA16 harbored the same mutations in *KRAS* as the primary tumors from which each model was derived (PA4 and PA16: G12D; PA10: G12R)^[25]^. We used the same approach to confirm that each gemR PDX model retained the *KRAS* mutation present in the drug-sensitive parent PDX model from which it was derived [Figure 6]. DNA from human Panc1 cells served as a positive control; DNA from mouse tissue was the negative control. The data show that DNA from the three pairs of parent and gemR models generated the expected 214 bp PCR product, and DNA from mouse tissue generated no PCR product [Figure 6A]. PCR products were excised, extracted, and sequenced. Sequencing data [Figure 6B] confirmed that gemR PDX tumors harbor the *KRAS* mutations of their respective parent models. The data document that the gemR PDX models are of human origin, and that they conserve the *KRAS* mutations present in drug-sensitive parent PDX tumors as well as in primary tumors of origin.

## Discussion

This study describes development of three PDAC PDX models (PA4.gemR, PA10.gemR, PA16.gemR) with gemcitabine resistance acquired *in vivo*, the first such models reported. Model development was based on the premise that models with resistance acquired *in vivo* would better reflect clinical characteristics and responses to gemcitabine than currently available models that are based on acquisition of resistance *in vitro*. We characterized these models for expression of proteins reported to contribute to gemcitabine resistance.

The timeframe for emergence of resistance differed among the three models, but all three were similar to their drug-sensitive counterparts in tumor cell morphology, degree of differentiation, and stroma:tumor cell ratio. All gemR models expressed higher levels of the cell proliferation marker Ki67 than gemcitabine-naïve controls (*P* < 0.001), suggesting that gemR models have a more aggressive phenotype^[50]^. No pattern of expression common to six proteins previously associated with gemcitabine resistance was evident among the models. Notably, the data demonstrate that changes in levels of expression of RRM1, hCNT1, hENT1, CDA and dCK were consistent with results in models with resistance acquired *in vitro* in only 2 of 8 changes observed. Data consistent with *in vitro* data were the increase in RRM1 in PA10.gemR tumors and the increase in CDA in PA4.gemR tumors. The multiple inconsistencies among models derived *in vitro* and *in vivo* suggest that resistance mechanisms likely differ, depending on the method of development. Further, no increase or decrease of a specific protein was always associated with another specific change, suggesting that each change occurs independently.

Previous work characterizing gemcitabine resistance in PDAC tumors has been done predominantly with human PDAC cell lines *in vitro*. While *in vitro* model systems have the advantages of being maintained in culture, providing opportunity for clonal expansion and genetic drift, the degree to which these cell lines mimic the clinical disease is controversial^[51,52]^. Bergman *et al*.^[53]^ described the first *in vivo* model of acquired gemcitabine resistance using murine Colon 26 tumors in 2005. The Colon 26 model is of murine origin and was developed from carcinogen-induced transplantable tumor system^[53-55]^. Models of human origin with resistance acquired *in vivo* to alkylating agents and olaparib have been reported for breast cancer^[56,57]^. These models are similar to those in this report in that individual models developed resistance at a unique rate.

The primary mechanism of gemcitabine resistance reported is the decreased expression or activity of dCK^[14]^. This decrease results in a reduction in the amount of active, phosphorylated gemcitabine and a decrease in dFdCTP available for incorporation into DNA^[14]^. In contrast to those data, in our gemR models dCK expression either remained unchanged or increased, indicating that loss of dCK expression is not a primary mechanism of resistance in these models. Interestingly, Bergman *et al.*^[53]^ also reported that in their murine colon tumor model with resistance to gemcitabine, dCK expression or activity was unchanged. Microarray data for that model did, however, reveal an increase in RRM1 expression^[53]^. In our models, RRM1 expression met the criteria for a > 1.5-fold in one of the three models. For the other five proteins reported to be associated with gemcitabine resistance, of particular note were changes that were counterintuitive. For example, PA4.gemR tumors expressed higher levels of hCNT1 (1.8-fold) and hENT1 (12.5-fold), rather than the expected decrease of these two membrane-bound transporters. PA10.gemR and PA16.gemR tumors also showed this apparent anomalous increase in expression of hENT1. In addition to the unexpected increase in hENT1 in PA10.gemR tumors, there was also a 3.3-fold increase in dCK expression. However, while PA10.gemR tumors show a 60% increase in RRM1 expression, we speculate that this would be unlikely to confer the level of resistance seen with this model, considering the simultaneous increases in hENT1 and dCK expression. We speculate further that the apparent anomalous 3.6- and 1.6-fold increases in hCNT1 and hENT1- the sole changes observed in PA16.gemR tumors - are also unlikely to account for the observed resistance phenotype in this model. We suggest that PDAC PDX models with gemcitabine resistance acquired *in vivo* harbor resistance mechanisms not previously identified, using models with resistance developed *in vitro*. Also, because overall no pattern of expression of the six marker proteins was evident in all three models, it is likely that each resistance mechanism develops independently. Overall, the data suggest that gemcitabine resistance acquired *in vivo* may depend on as-of-yet unidentified mechanisms.

In summary, our study identified no consistent changes in any one protein reported to be associated with gemcitabine resistance common to all three PDAC PDX gemR models. This observation suggests that additional mechanisms for gemcitabine resistance acquired *in vivo* remain to be identified. Current work in our lab focuses on identifying mechanisms of resistance in these models that, potentially, impact carbohydrate or lipid metabolism and to identify drug targets relevant to clinical disease.

## Declarations

### Copyright

© The Author(s) 2020.
